# The effect of palmitic acid on inflammatory response in macrophages: an overview of molecular mechanisms

**DOI:** 10.1007/s00011-019-01273-5

**Published:** 2019-07-30

**Authors:** Jan Korbecki, Karolina Bajdak-Rusinek

**Affiliations:** 1 Department of Molecular Biology, School of Medicine in Katowice, Medical University of Silesia, Medyków 18 St., 40-752 Katowice, Poland; 2 Department of Medical Genetics, School of Medicine in Katowice, Medical University of Silesia, Medyków 18 St., 40-752 Katowice, Poland

**Keywords:** Palmitic acid, Saturated fatty acid, Obesity, Inflammation, Insulin resistance, Macrophage

## Abstract

Palmitic acid is a saturated fatty acid whose blood concentration is elevated in obese patients. This causes inflammatory responses, where toll-like receptors (TLR), TLR2 and TLR4, play an important role. Nevertheless, palmitic acid is not only a TLR agonist. In the cell, this fatty acid is converted into phospholipids, diacylglycerol and ceramides. They trigger the activation of various signaling pathways that are common for LPS-mediated TLR4 activation. In particular, metabolic products of palmitic acid affect the activation of various PKCs, ER stress and cause an increase in ROS generation. Thanks to this, palmitic acid also strengthens the TLR4-induced signaling. In this review, we discuss the mechanisms of inflammatory response induced by palmitic acid. In particular, we focus on describing its effect on ER stress and IRE1α, and the mechanisms of NF-κB activation. We also present the mechanisms of inflammasome NLRP3 activation and the effect of palmitic acid on enhanced inflammatory response by increasing the expression of FABP4/aP2. Finally, we focus on the consequences of inflammatory responses, in particular, the effect of TNF-α, IL-1β and IL-6 on insulin resistance. Due to the high importance of macrophages and the production of proinflammatory cytokines by them, this work mainly focuses on these cells.

## Introduction

In developed countries, an overweight and obesity is a growing epidemiological problem. It is estimated that in the North America and Europe nearly 60% of the population is overweight and 15% are obese [1–3]. This percentage is increasing steadily for over 30 years.

Obese patients have significantly increased free fatty acid (FFA) levels in the blood [4–8]. The FFA includes palmitic acid (PA) and other fatty acids such as stearic acid, monounsaturated fatty acids (MUFA) (oleic acid) and polyunsaturated fatty acids (PUFA) (linoleic acid) [8, 9]. Consuming large amounts of saturated fatty acids (SFA), in particular PA, and metabolism disorders, increases the concentration of these fatty acids in the blood. This leads to inflammatory responses, which are an important factor in the development of diseases associated with obesity, for instance, insulin resistance [10].

PA induces inflammatory responses; however, it does so by activating different signaling pathways. Some pathways may interact, while others may only occur in specific cells. This work focuses mainly on macrophages, due to their high importance and role in proinflammatory cytokines production.

## The impact of the palmitic acid on the cell functions

When PA gets into the cell, it is metabolized to saturated phospholipids (mainly to lysophosphatidylcholine) [11–14], diacylglycerol (DAG) [11, 15–18] and ceramides [17, 19–23].

In general, fatty acids such as MUFA are metabolized and then accumulated in the form of low-toxic triacylglycerol (TAG). However, a large amount of PA inhibits the TAG synthesis at the DAG stage, which is then accumulated in the cell. The exact mechanism of this process is still poorly understood. Probably the diglyceride acyltransferase (DGAT), an enzyme involved in the synthesis of TAG from DAG and acyl-CoA, has less activity when the substrates are saturated DAG and saturated acyl-CoA [20, 24, 25]. Another possible explanation is that PA induces the production of reactive oxygen species (ROS), which inhibits the DGAT2 activity [26].

Palmitate is mainly incorporated into DAG because its incorporation into TAG is reduced by the fall in the expression of DGAT2. It was shown that after 12 h incubation with 150 μM of PA, the DGAT2 mRNA expression was reduced in the murine proximal tubular epithelial cell model [27] and after 16 h of incubation with 500 μM PA in murine C2C12 myoblasts [28]. However, after 24-h incubation with 300–900 μM PA, the expression of DGAT1 and DGAT2 increased in anserine primary hepatocytes [29]. In turn, when they treated those hepatocytes with even higher concentration of PA, DGAT1 and DGAT2 expression started to decrease to the control level.

In addition to DGAT1 and DGAT2, PA does not change the activity of diacylglycerol kinases involved in DAG metabolism, which was shown in vascular smooth-muscle cells [30]. Further, DAG activates protein kinases C (PKC), which is important in TLR2 and TLR4 activation, as well as in the activation of nuclear factor κB (NF-κB).

It is known that DAG is the activator of conventional PKC (cPKC) and novel PKC (nPKC). However, different DAGs may activate different PKCs [31]. For example, PKCδ is poorly activated by 16:0/16:0-DAG, but strongly by other DAGs containing unsaturated fatty acids in its structure. In turn, PKCε is poorly activated by all DAGs. PKCθ, on the other hand, is strongly activated by all types of DAGs. In addition to the activation, also palmitoyl-CoA can cause acylation of PKC, which increases the activity of the kinases of this group [32]. However, DAG does not activate PKCζ [33]. Activators for this kinase are ceramides [34, 35]. Nevertheless, the importance of individual PKCs depends on the level of its expression in the tissue. PKCζ and PKCδ expression occurs in all tissues [36]. The expression of PKCε occurs mostly in the brain, kidneys and heart, and in other tissues, it is very low [36, 37]. In contrast, PKCθ expression is specific for muscle but is not expressed in adipocytes, macrophages or liver cells [38].

**Fig. 1:**
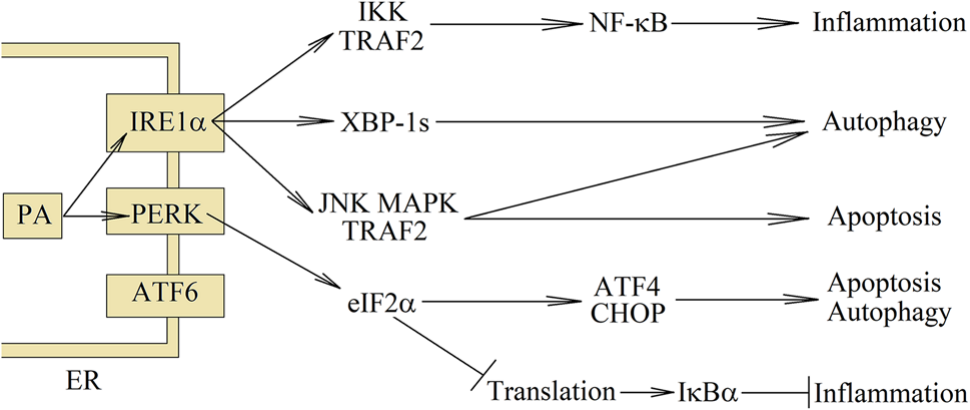
The consequences of PA-induced ER stress. At high concentrations, PA is converted to saturated lysophosphatidylcholine and DAG, which are incorporated into the ER. This causes ER stress and activation of ER stress sensors: IRE1α and PERK. The same pathways are activated during the detection of unfolded proteins. In particular, eIF2α phosphorylation, represses the translation of many genes with the exception of few, such as CHOP or ATF4. Then NF-κB is activated, which leads to apoptosis suppression and induction of inflammatory reactions. Activation of ER stress sensors is involved in increasing the capacity to maintain autophagy in stressed cells; however, severe ER stress leads to cell apoptosis

The PA, along with PKC activation, can also affect the endoplasmic reticulum (ER) stress (Fig. 1). PA is metabolised to phospholipids (mainly to lysophosphatidylcholine) and to the DAG. Both of these substances contain saturated hydrocarbon chains. High concentration of PA leads to saturated DAG and saturated lysophosphatidylcholine accumulation in the ER [14, 18, 39], which causes destructive changes in its structure. These changes are detected by transmembrane domain of inositol-requiring enzyme 1α (IRE1α) and protein kinase RNA-like endoplasmic reticulum kinase (PERK) but not by activating transcription factor (ATF)6 [40–42]. This leads to the activation of ER stress sensors; however, under the influence of saturated lipids, there is no formation of large cluster with IRE1α, but only dimerization of this ER stress sensor. IRE1α is a kinase and endonuclease that catalyzes the excision of an intron from X-box-binding protein-1 (XBP-1) mRNA to form splice XBP-1 (XBP-1s) [14, 43].

PERK, on the other hand, is a kinase that phosphorylates eukaryotic initiation factor 2α (eIF2α), what causes the repression of translation. Nevertheless, PERK activation by ER stress increases translation of C/EBP homologous protein (CHOP) and ATF4 and then CHOP- and ATF4-dependent autophagy genes, which are involved in increasing the capacity to maintain autophagy in stressed cells [14, 43–47]. Autophagy also depends on c-Jun NH2-terminal kinase (JNK)–mitogen-activated protein kinase (MAPK). Autophagy is a key mechanism to protect the cell against lipotoxicity. Nevertheless, the prolonged exposure of the cells to the lipotoxic environment causes the mammalian target of rapamycin (mTOR)-dependent autophagy inhibition [48] and leads to cell apoptosis. CHOP is involved in apoptosis induction in hepatocytes [49, 50]. Although activation of JNK–MAPK by glycogen synthase kinase-3β (GSK-3β) is also important in the induction of apoptosis [12, 49, 51, 52], the exact mechanism of GSK-3β activation through PA is still unclear. It can not only be activated by the ER stress, but also independently of the ER. Despite apoptosis induction, ER stress is important in the induction of inflammatory response [53, 54]. It activates NF-κB and NOD-like receptor pyrin domain containing 3 (NLRP3) inflammasome, which results in increased production of proinflammatory cytokines.

Various functions of mitochondria are strongly affected by PA treatment. Normally, ROS generation is relatively low, but in the presence of PA it increases significantly. This increase is mostly due to the partial inhibition of complexes I and complexes III of the respiratory chain [55–58]. Another effect of PA is the inhibition of the mitochondrial adenine nucleotide translocator activity, which causes accumulation of ATP in these organelles and increased production of ROS [59, 60]. ROS functions as specific second messenger that participates in the induction of inflammatory response, for example, it may trigger the activation of NF-κB.

The other lipids synthesized from PA are ceramides. Their increased amount causes an up-regulation in the expression and increased activity of neutral sphingomyelinase (nSMase) and serine palmitoyltransferase (SPT) [23]. Probably, it happens through the activation of NF-κB and increased expression of its downstream genes [61] or through the ER stress, which activates the IRE1α => XBP-1s pathway [62]. This results in an increased de novo synthesis of ceramides as well as increased release of sphingosine from the cell membrane, which is transformed into ceramides. Also, incubation of the cells with PA causes the accumulation of palmitoyl-CoA, which is a substrate for ceramide production. It seems that the production of de novo ceramides under the influence of PA is important for enhancing the signal transduction through TLR4 [21, 22].

## Macrophages in obesity

One of the molecular symptoms of obesity is the occurrence of chronic low-grade inflammation. Macrophages play an important role in these processes in adipose tissue [63–65] and to a lesser extent in the liver [66]. This immune system cells can be tissue-resident macrophages such as microglia in the brain and Kupffer cells in the liver or be recruited into the tissues from the blood monocytes. In this process, monocytes differentiate and then polarize into specific macrophage phenotypes, depending on the factors acting on these cells [67]. There are two major macrophage sub-populations with different functions: inflammatory M1 and anti-inflammatory M2 macrophages. Functionally, the M1 macrophages produce pro-inflammatory cytokines and participate in the removal of pathogens and cancer cells. In turn, M2 macrophages produce anti-inflammatory cytokines, e.g., interleukin (IL)-10 and participate in the remodeling of the tissue during wound healing, regulation of the immune system and dampening of inflammation.

In lean animals and healthy people, resident macrophages in adipose tissue are polarized toward anti-inflammatory M2 state [64, 68–70]. Nevertheless, in obesity, adipocytes and these adipose tissue-resident macrophages produce chemokines that cause the recruitment of pro-inflammatory macrophages [64, 69–72]. This is a C–C motif chemokine receptor (CCR)2, CCR5, CCR7 and C–X3–C motif receptor 1 (CX3CR1) chemokine-dependent process. These pro-inflammatory macrophages localize in the environment of necrotic adipocytes, forming so-called crown-like structures [64, 71, 73] and start to produce pro-inflammatory cytokines [68]. Nevertheless, in humans, these macrophages have mixed phenotype [74]. The surface marker expression (integrin αvβ5, CD163, CD200, CD206, CD209, CD1b and CD1c) on these cells is similar to that on M2-polarized macrophages. At the same time, they show very high basal production of pro-inflammatory cytokines, even higher than in M1 macrophages. This mixed phenotype is associated with various factors that affect these macrophages. In adipose tissue, cell debris and free lipid droplets from necrotic adipocytes enhance inflammatory reactions and polarization of M1 macrophages [71, 73, 75]. Also in obesity, adipose tissue hypoxia induces pro-inflammatory M1 activation [76, 77]. Other pro-inflammatory factors affecting macrophages in advanced obesity are hyperglycemia [78, 79] and hyperinsulinemia [80, 81]. Nevertheless, in the described macrophages, there is a high expression of the peroxisome proliferator-activated receptor (PPAR)γ, which limits inflammation [82, 83].

## Palmitic acid is a toll-like receptor agonist

In many studies, where specific inhibitors and siRNAs have been used, the results showed that TLR2 and TLR4 are activated by SFA, such as PA [61, 84–92] and lauric acid [93, 94]. The effect was especially visible in cells incubated for more than 12 h with a given SFA.

**Fig. 2:**
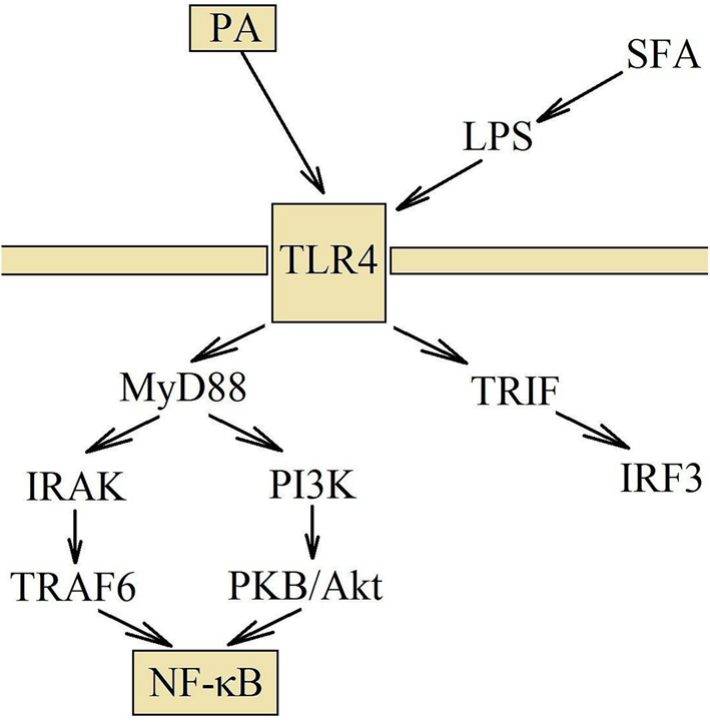
TLR4 activation via PA and signal transduction. PA activates TLR4 directly, but it can also activate this receptor indirectly. Consuming large amounts of fats causes disorder in intestinal functions, which leads to increased amount of LPS in the blood. After TLR4 activation, the signal transduction takes place via the IRAK and PI3K =>PKB/Akt pathways. They lead to the activation of NF-κB and the induction of inflammatory reactions

PA induces the activation and dimerization of TLR2 with TLR1, TLR2 with TLR6 or TLR4 [88, 90]. After TLR4 or TLR2 activation, the receptor dimerization and recruitment to lipid rafts take place. This is followed by signal transduction through MyD88 and NADPH oxidase activation [93–95]. The signal is transmitted through two pathways, the myeloid differentiation factor 88 (MyD88) => IL-1 receptor-associated kinase (IRAK) => TNF receptor-associated factor (TRAF)6 and the phosphatidylinositol 3-kinase (PI3K) => protein kinase B (PKB)/Akt pathway [93, 94]. As a consequence, it activates NF-κB. However, also the SFA-mediated TLR4 activation may initiate the MyD88-independent signaling pathway: TLR4 => IFNβ-mediated transcription factor (TRIF) => interferon regulator factor (IRF)3 (Fig. 2) [94]. The signaling mechanism induced by SFAs is mediated by TLR4, but activation of TLR4 requires complex formation with an accessory protein called myeloid differentiation protein 2 (MD2). PA, as well as natural TLR4 agonist, LPS, associates with the hydrophobic binding pocket of this TLR4 adaptor protein MD2, which activates signal transduction [96, 97].

Notably, a number of groups have independently proposed that SFAs, including PA may also indirectly stimulate TLR-dependent signaling [98–100], especially after a very short exposure time of the cells to a given SFA [98]. Moreover, PA treatment at either time point induces only 8% of the genes induced by LPS [54]. Molecular simulation of PA interactions with TLR4-MD2 also questions whether PA is an agonist of this receptor [100]. In turn, some papers showed the TLR4-dependent effect of PA on inflammatory responses, but at a different time point than the effect of its agonist, LPS alone [101, 102]. This may indicate the indirect PA activation of TLR4, by increasing the production of some noncanonical TLR4 activators. The explanation may also be that PA enhances the signal transduction, or that TLR4 forms complexes with other receptors, e.g., with cluster of differentiation 36 (CD36), through which TLR4 may be activated by PA [103]. Nevertheless, it is possible that PA may directly activate TLR4, as well as indirectly by inducing the same signaling pathways causing ER stress and generation of DAGs and ceramides in the cell.

### Palmitic acids produce activators for toll-like receptors

In addition to LPS, other substances can also activate TLR4. For example, extracellular ceramides in electronegative LDL or extracellular histones. Electronegative LDL is the LDL fraction whose blood levels are elevated in obese people [104]. Electronegative LDL presents a PLC-like activity [105], which is related to high ceramide content. This is important in inducing inflammatory reactions through CD14 and TLR4 in macrophages and monocytes [106–108]. Nevertheless, the incubation of hepatocytes with PA causes the release of very similar particles: extracellular vesicles, which contain ceramides. The hepatocyte exposure to PA causes the ER stress. It activates the IRE1α => XBP-1s pathway and thus increases the expression of SPT1. This results in increased de novo ceramide production, which is secreted in extracellular vesicles outside the cell [62]. The formation of the extracellular vesicles also depends on IRE1α activation. Extracellular vesicles, which contain ceramides, may be pro-inflammatory; however, this hypothesis requires confirmation and careful research.

Another possible mechanism for activating TLR by PA is the increase of extracellular histone release. It was already shown that PA induces the release of histone H3 from activated macrophage RAW 264.7 cell line [109], but this process is not related to cell death. PA induces the release of histone H3 from macrophages, in part, through the ROS generation and the JNK–MAPK signaling pathway [109]. Extracellular histones directly bind and activate TLR2 [110–113], TLR4/MD2 [110–113] and TLR9 [114]. Although histones in the complex with DNA activate TLR much better than histones alone [111], this TLR activation causes MyD88-dependent activation of NF-κB and thus increases the production of proinflammatory cytokines. Extracellular histones induce the expression of adhesion molecules, intracellular adhesion molecule-1 (ICAM-1) and vascular cell adhesion molecule-1 (VCAM-1), in endothelial cells [109]. This results in the trafficking of monocytes and macrophages across the vessel wall and recruitment of these cells to the tissues. However, it is postulated that extracellular histones are actually a component derived from neutrophil extracellular traps (NET) [115]. NET is the process by which immune cells, mainly neutrophils, defend the organism against pathogens. It involves the release of the cell nucleus content or mitochondrial DNA, outside of the cell. Due to the fact that histones are proteins associated with DNA, in this process, they are removed out of the cell as well.

### Indirect effect of palmitic acid on toll-like receptor 4 activation: increase in lipopolysaccharide levels

PA may indirectly act on TLR4. In particular, it may help to activate this receptor by increasing the amount of LPS in the blood. High lipid concentration in the intestines causes impairment in intestinal barrier function [116, 117]. This facilitates the passage of bacteria and LPS through the intestinal wall. LPS goes to chylomicrons and through the portal vein enters the bloodstream [118]. At the same time, in obese and diabetic people, there are changes in the composition of gut microbiota, which affect the function of the intestines and the amount of LPS entering the blood [116, 119, 120]. Hence, consuming food with a large amount of SFA, and with a small amount of fiber and 3-n PUFA, causes an increase of LPS concentration in the blood [121–124]. Also people with obesity, atherosclerosis or type 2 diabetes mellitus (T2DM) have increased concentration of LPS in their blood [121, 125–128]. Consumption of large amounts of PA results in increased levels of PA and LPS in the blood. Due to the fact that LPS is a TLR4 agonist, large amounts of PA may indirectly activate TLR4.

## Indirect effect of palmitic acid on toll-like receptors activation

In the cells, PA can act in different ways, activating many TLR-dependent signaling pathways. As a consequence, TLR can be repeatedly activated during multi-day treatment of the cells with this SFA [87]. PA may increase the mRNA expression and protein levels of TLR4, which enhances the signal transduction of this receptor [129–131]. It can also support the activation of TLR4. After activation by LPS, TLR4 is translocated into the lipid raft to assemble the complex involved in signal transduction. At this first stage, most important is the composition of lipids in the cell membrane, in particular, endogenous cholesterol synthesis. Thanks to the fact that PA, the same as LSP, increases the expression of the fatty acid synthase (FAS), and thus increases production of substrates for the production of cholesterol and facilitates the activation of TLR4 [132, 133].

### Role of protein kinase C in toll-like receptor-mediated signaling pathway

After LPS-mediated TLR4 activation, a signal transduction occurs. This results in the activation of NF-κB and an increased production of proinflammatory cytokines. PKC is involved in the transmission of this signal. PKCζ is important in the translocation of TLR4 into the lipid rafts and NF-κB activation, which was proofed in the myometrial cell model [134], THP-1 macrophages [135] and human peripheral blood monocytes and macrophages [136]. After the activation of TLR4, PKCζ is activated by RhoA. Next, the PKCζ activates the transforming growth factor beta-activated kinase (TAK)1, which is then involved in the activation of NF-κB. Identical signal transduction occurs via TLR2 [137]. However, experiments on RAW264.7 macrophages showed that TLR2 and TLR4 activate PKCδ which then binds to Toll–Interleukin 1 Receptor Domain Containing Adaptor Protein (TIRAP)/Mal. This is important in the p38 MAPK and NF-κB activation [138]. Also in the same macrophages, activation of TLR4 (through the MyD88-depending pathway) causes binding and phosphorylate of PKCε, which is important in the activation of NF-κB [139]. Because PA increases the concentration of PKC activators: DAG and ceramides, it can enhance, by synergy effect, the TLR4- and TLR2-mediated signaling pathways [22, 140, 141].

### Significance of endoplasmic reticulum stress in toll-like receptor-mediated signaling pathway

**Fig. 3:**
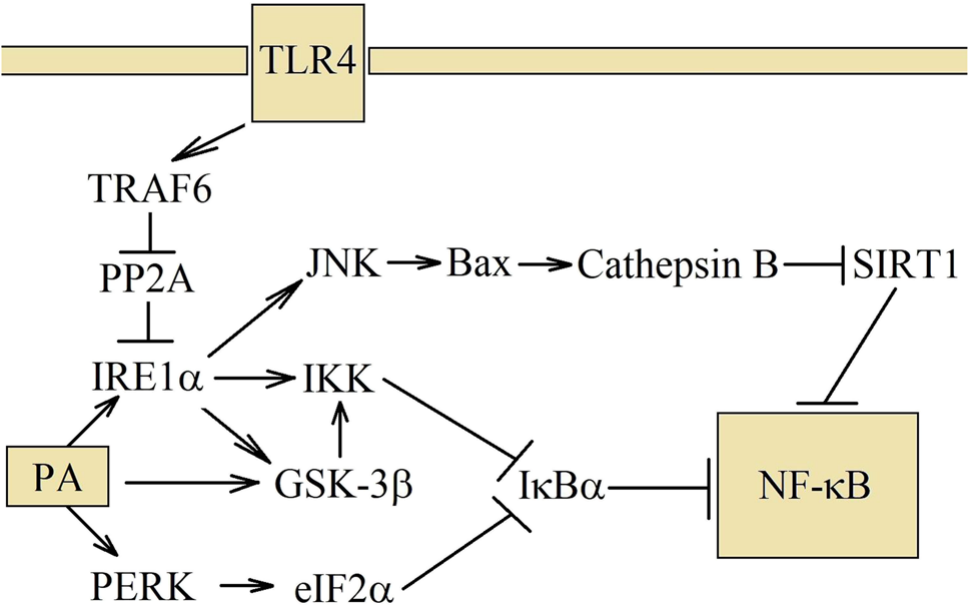
Mechanism of NF-κB activation by PA. PA and TLR4 share some of the signaling pathways. Both activates IRE1α, but in a different way. TLR4 activates this ER stress sensor via TRAF6. In turn, activation through PA depends on the damage of ER membranes and incorporation into them. Then IRE1α activates JNK–MAPK pathway, which destabilizes the lysosomes. Cathepsin B is released, which is involved in the NF-κB activation. IRE1α also activates IKK, which participates in the canonical activation of NF-κB. PA can also cause activation of PERK, which inhibits translation and thereby reduces the level of IκBα that leads, as well, to the NF-κB activation

One of the ER stress sensors activated by TLR is IRE1α. Interestingly, TLR does not activate other ER stress sensors, such as ATF6 nor PERK [142]. TRAF6 plays a key role in TLR-mediated IRE1α activation by catalyzing IRE1α ubiquitination and blocking the recruitment of protein phosphatase 2A (PP2A) (Fig. 3) [143]. Phosphorylation of IRE1α and thereby increased activation of this protein result in the XBP-1 mRNA splicing, which results in the creation of XBP-1s. Activation of this pathway does not cause the expression of the ER stress proteins, or even decrease the activation of ATF6 and PERK. The consequence of TLR-induced IRE1α activation is the production of proinflammatory cytokines [53, 142, 143], in particular IL-1β and CC motif chemokine ligand (CCL)5/regulated on activation, normal T cell expressed and secreted (RANTES) and, in part, tumor necrosis factor-α (TNF-α).

SFA treatment also displays ER stress and activates the ER stress sensor, IRE1α. This, in turn, activates NF-κB and increases IL-1β production [53, 54]. However, in this model, TNF-α expression is, in part, dependent on the IRE1α =>XBP-1s pathway.

Unlike TLR, PERK can be activated by SFA, which may be an argument that SFAs do not act as TLR agonist, but they induce signaling pathways with the same effect as TLR activation [54, 142, 143], although, in the hepatocyte HepG2 cell line, activation of IRE1α by PA is TLR4 dependent [144]. Most probably, PA participates in two processes simultaneously. First, it integrates with the ER, causing the activation of IRE1α and PERK, and second, induces TLR4-mediated IRE1α activation.

### Activation of nuclear factor κB and NOD-like receptor pyrin domain containing 3 inflammasome

Two factors play an important role in increasing IL-1β production: increased expression of pre-IL-1β, and activation of inflammasome. In the latter, the pre-IL-1β proteolysis to IL-1β occurs.

**Fig. 4:**
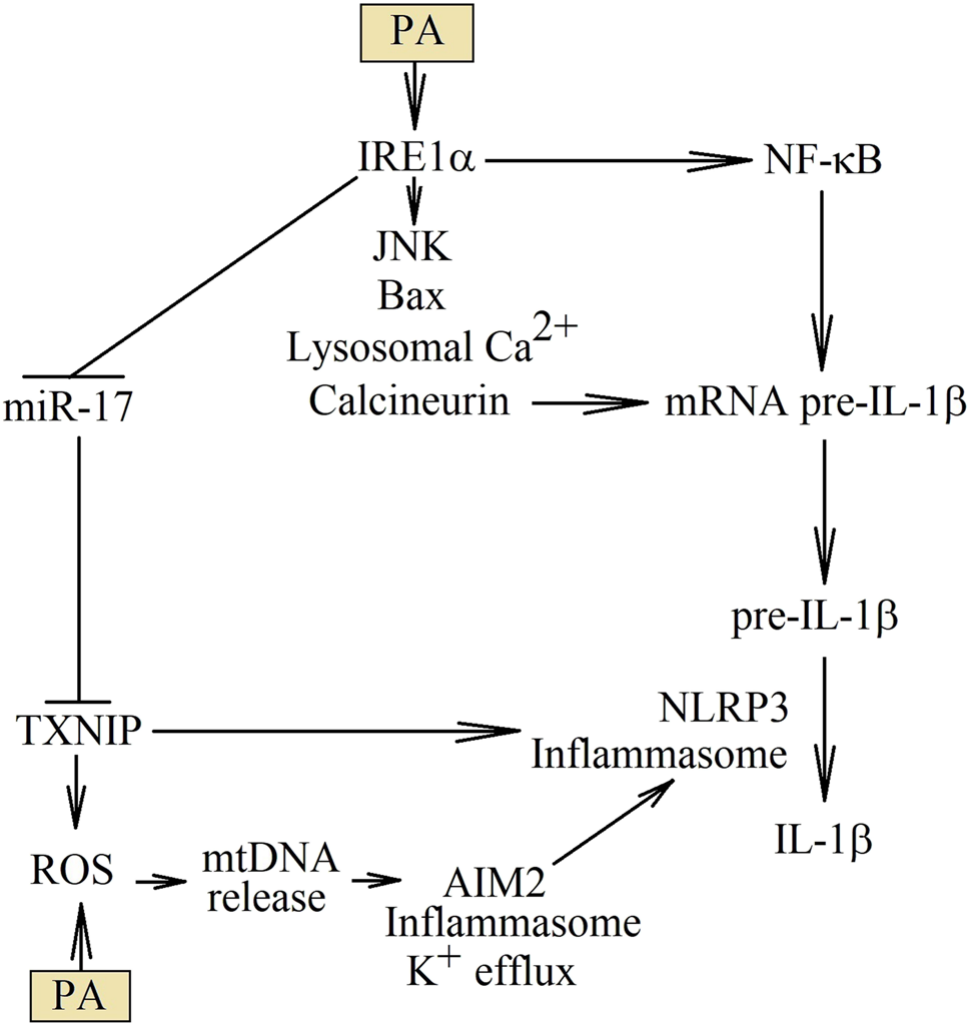
PA results in increased production of IL-1β. PA increases the production of IL-1β at various levels of this cytokine synthesis. First, PA activates NF-κB, which increases the expression levels of pre-IL-1β mRNA. Second, PA can increase the stability of this transcript by destabilizing lysosomes, releasing from them the Ca^2+^ ions and thereby activating calcineurin. Finally, PA activates NLRP3 inflammasome, which is associated with increased TXNIP expression, or released mitochondrial DNA to the cytoplasm

ER stress and activation of IRE1α are responsible for the production of IL-1β in macrophages under the PA treatment (Fig. 4) [53, 54]. NF-κB activation is responsible for the increase of pre-IL-1β expression. During the ER stress, activated IRE1α forms a complex with inhibitor of NF-kB (IκB) kinase (IKK) and TRAF2, which results in the activation of IKK and subsequent activation of NF-κB [145]. In turn, PERK phosphorylates eIF2α, which represses the translation of certain genes including IκBα [146]. IκBα is a protein with a short half-life. With repressed translation, the amount of IκBα is reduced and hence the activation of NF-κB. In the ER stress, activation of GSK-3β results in increased production of IL-1β [53]. Nevertheless, the role of GSK-3β in inflammatory responses induced by PA is still unclear. Probably this kinase phosphorylates IKKγ/NEMO, which causes activation of NF-κB [147].

The ER stress induced by PA causes inflammatory responses through the destabilization of lysosomes. This happens by translocating Bax to lysosomes and destabilizing the lysosomal membrane [148–150]. It may depend on the JNK–MAPK activation [49, 151]. However, the mechanism of JNK–MAPK activation by PA is still unknown. It may also depend on IRE1α [152] or be activated by GSK-3β, independently of the ER stress [12]. Without a doubt, the process of lysosomes destabilization is independent of ceramides and cathepsin B. Destabilization of the lysosome membrane releases cathepsins into the cytoplasm. When this proteases, including cathepsin B, can perform the sirtuin (SIRT)1 proteolysis and the activity of this NF-κB deacetylase is decreased. As a consequence, the acetylation increases and thus the activation of p65 NF-κB [153]. This pathway is also known to be important in the activation of TLR4-mediated NF-κB signaling.

As shown by the experiments on high-fat choline-deficient food-fed mice, the activation of NLRP3 inflammasome alone may not depend on cathepsin B [153]. Even more, in macrophages, activating NLRP3 inflammasome can destabilize lysosomes [154]. Nevertheless, mainly it is possible to activate NLRP3 inflammasome by cathepsin B, as demonstrated by experiments on microvascular endothelial cells treated with PA [155]. In addition to cathepsins, also the Ca^2+^ ions are released from lysosomes, which causes the activation of calcineurin, increases the stability of pre-IL-1β mRNA and increases the expression of this polypeptide [156].

However, the PA may trigger the activation of NLRP3 inflammasome through other mechanisms. For example, PA damages the mitochondria, by reducing membrane potential and increasing ROS production. This results in mitochondrial DNA release into the cytoplasm [157], which leads to an activation of AIM2 inflammasome [158]. As a consequence, there is a cell membrane perforation, efflux of K^+^ ions and activation of NLRP3 inflammasome.

Another way to activate NLRP3 inflammasome is the PA-induced ER stress, leading to thioredoxin interacting protein (TXNIP) protein expression [159, 160] Activation of IRE1α reduces the expression of miR-17 which normally destabilizes TXNIP mRNA. Thanks to this, PA increases the expression of TXNIP protein by inducing ER stress. In the mitochondria, TXNIP binds and disturbs the action of thioredoxin H-type 1 (TRX1), and increases the generation of ROS. ROS is involved in the activation of NLRP3 inflammasome [161]. Moreover, TXNIP can directly bind to the NLRP3 inflammasome, which results in the activation of this inflammasome and the formation of IL-1β [161].

## Fatty acid-binding protein 4/aP2 and peroxisome proliferator-activated receptor γ

**Fig. 5:**
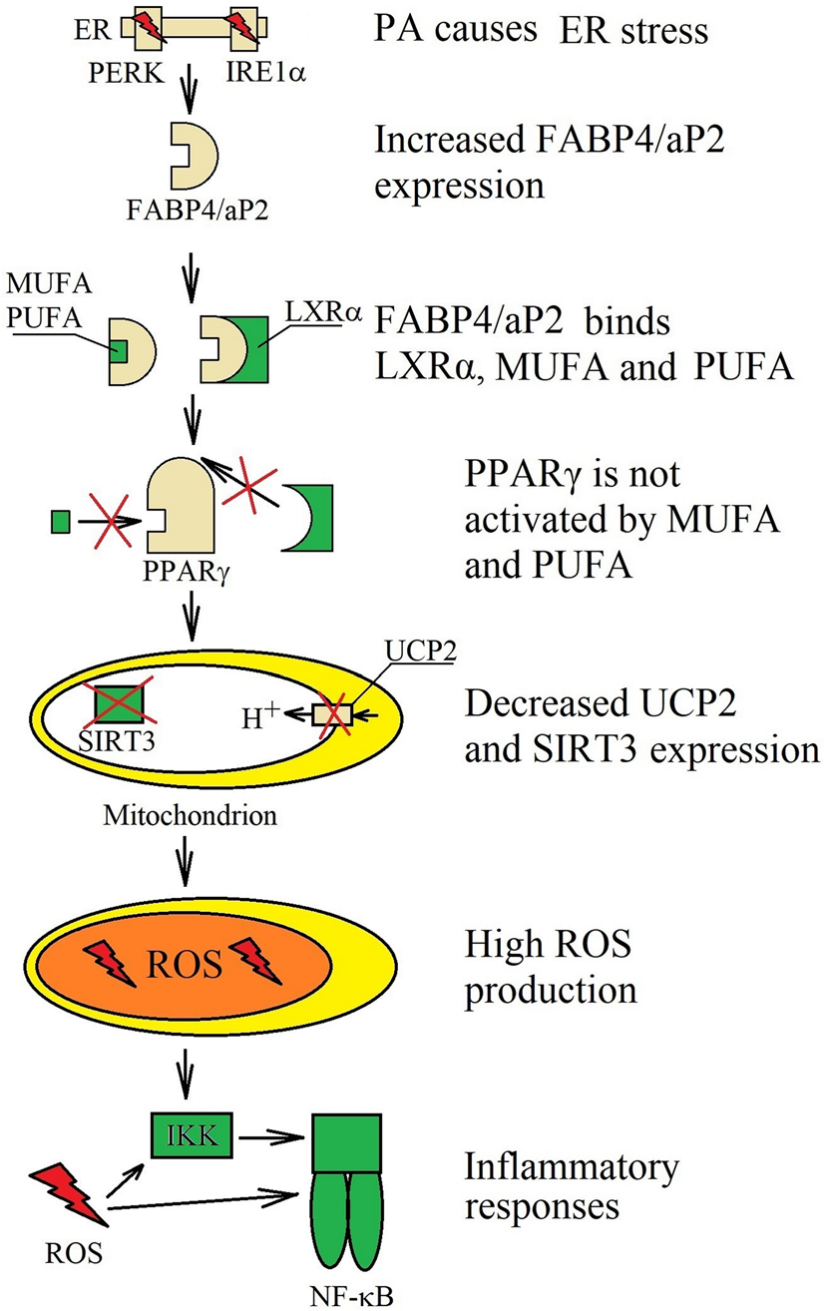
Role of FABP4/aP2 in PA activity. PA-induced ER stress increase the expression of FABP4/aP2. It is a protein that binds MUFA and PUFA that prevents the activation of PPARγ. FABP4/aP2 also binds LXRα, disrupting the expression of PPARγ-dependent genes. This reduces the expression of SIRT3 and UCP2, which in turn results in increased generation of ROS that is involved in inflammatory responses. Moreover, PPARγ inhibits NF-κB activation; therefore, functional disorders in PPARγ, results in increased activation of NF-κB

Another important role of PA-induced inflammatory response is the increase of fatty acid-binding protein 4 (FABP4)/aP2 expression (Fig. 5). In macrophages, the PA increases the expression of FABP4/aP2 protein, but not FABP4/aP2 mRNA [162, 163] and this process is related to the ER stress induction. In turn, LPS increases mRNA and protein levels of FABP4/aP2 found in macrophages, which shows that PA does not affect the expression of this protein via TLR4, only through the ER stress induction [164]. The FABP4/aP2, another name for adipocyte fatty acid-binding protein (A-FABP), is a fatty acid-binding protein. Nevertheless, unlike other FABPs, FABP4/aP2 has the same affinities to PA as to oleic acid and docosahexaenoic acid (DHA) [165]. As a result, the concentration of free MUFA and PUFA in the cells is reduced by FABP4/aP2 [166].

Increased FABP4/aP2 expression by PA, results in the lower expression of many proteins, including SIRT3, uncoupling protein 2 (UCP2) and peroxisome proliferator receptor-γ coactivator 1 (PGC-1)α, which interferes with mitochondrial function [163, 166–168]. This leads to increased ROS generation and increased inflammatory responses. This has been confirmed in C2C12 skeletal muscle cells where palmitate reduces PGC-1α expression through a mechanism involving NF-κB activation [169].

FABP4/aP2 binds LXRα, MUFA and PUFA which results in the reduction of the expression of LXRα-dependent and PPARγ-dependent genes [170]. PPARγ is a transcription factor and a nuclear receptor which is activated by MUFA and PUFA. Inhibiting the function of PPARγ reduces the expression of ATP-binding cassette subfamily A member 1 (ABCA1) and ATP-binding cassette subfamily G member 1 (ABCG1), proteins involved in the clearance of cholesterol from macrophages [162, 171]. Also, the stearoyl-coenzyme A desaturase (SCD) expression, which is SFA processing desaturase, protecting from negative effects of PA, is inhibited. Inhibition of PPARγ enhances the activity of IKK and NF-κB [170] and reduces the expression of CD36 [170, 172]. However, it should be noted that PA increases the expression of CD36 by inducing the ER stress [43, 83, 103, 173].

The experiments on macrophages showed that the expression of ABCA1 and ABCG1 is increased in the cells treated with PA [83, 174]. This is related to the other effect caused by PA, in particular, the ER stress and increased PPARγ expression. Probably, increased PPARγ expression depends on the ER stress activation of the IRE1α =>XBP-1s pathway [175].

FABP4/aP2 can also decrease the expression of UCP2. It is an uncoupling protein that reduces the generation of ROS in the mitochondria. However, increasing ROS production by PA treatment results in increased expression of UCP2 [45, 168, 176–178]. Nevertheless, increased expression of FABP4/aP2 in macrophages abolishes this effect, or even reduces the expression of UCP2, which enhances the generation of ROS and induces inflammatory responses [166, 178]. The reduced expression of UCP2 is due to reduced PPARγ activation [168].

Another protein whose expression in macrophages is reduced under the influence of FABP4/aP2 is SIRT3 [166]. It is a mitochondrial protein that causes deacetylation of superoxide dismutase (SOD)2, which increases the activity of this antioxidant enzyme and thus reduces the concentration of ROS [179]. Reduction in SIRT3 expression results in decreased activity of SOD2 which leads to increased generation of ROS in the mitochondria.

## Free fatty acid receptor 1/G protein-coupled receptor 40 plays a role as a receptor for palmitic acid in inflammatory responses

**Fig. 6:**
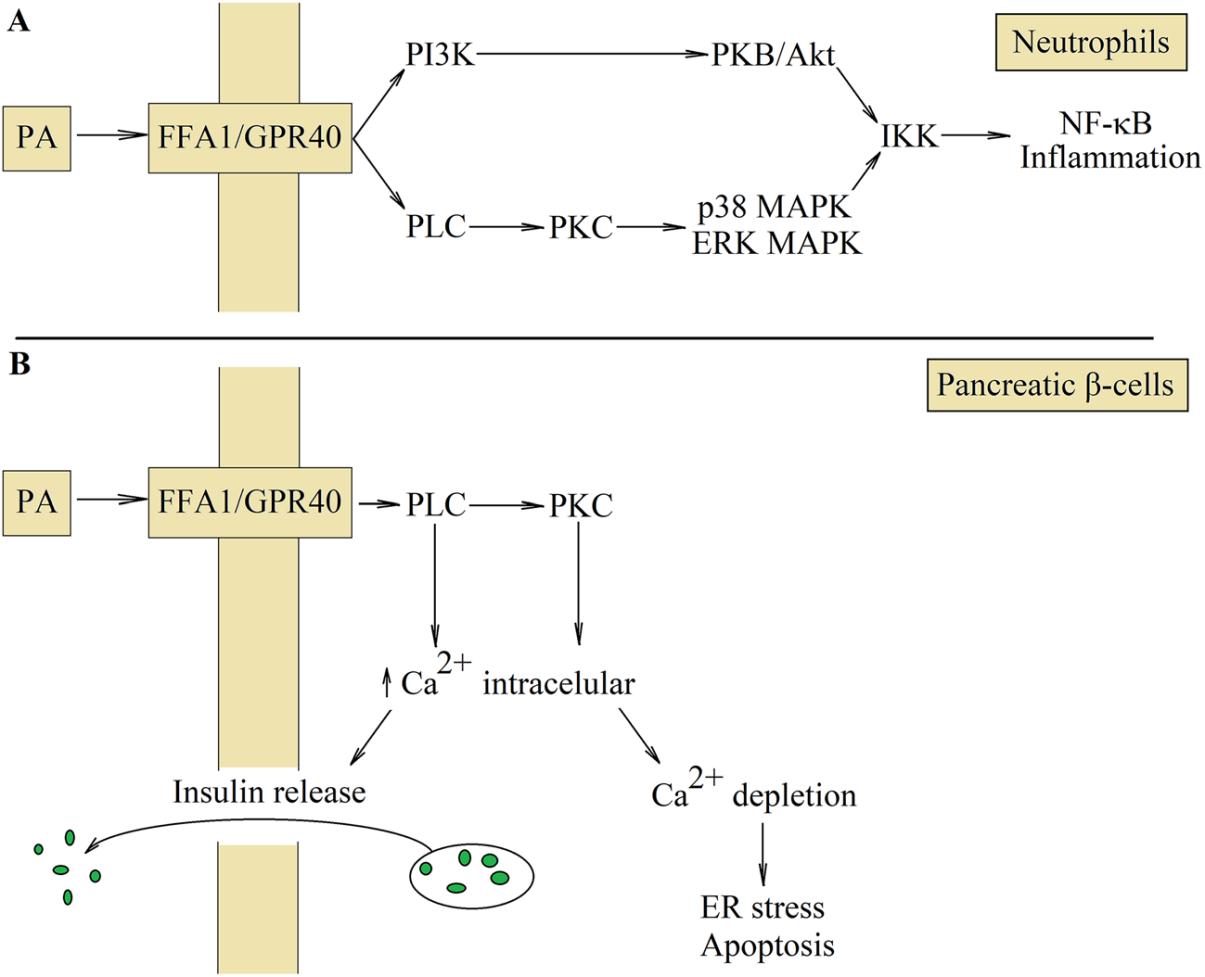
Activation of FFA1/GPR40 receptor. FFA1/GPR40 is the PA receptor whose activation enhances inflammatory reactions in neutrophils (**a**). This receptor causes signal transduction through PLC and PI3K, which activates IKK and consequently NF-κB. This transcription factor is involved in inflammatory reactions; however, activation of FFA1/GPR40 in pancreatic β-cells results in the release of insulin (**b**). Activation of PLC and PKC causes the release of Ca^2+^ from the ER to the cytoplasm. Higher cytoplasmic concentration of Ca^2+^ leads then to insulin release by pancreatic β-cells. However, continuous activation of FFA1/GPR40 results in Ca^2+^ depletion from ER and consequently the ER stress and pancreatic β-cells apoptosis

In addition to the presented way of action, PA may also activate its own free fatty acid receptor 1 (FFA1)/G protein-coupled receptor 40 (GPR40) receptor, which enhances inflammatory response (Fig. 6).

The highest expression of FFA1/GPR40 occurs in the brain, pancreas and monocytes, least in muscles, liver and adipose tissue [180]. Activation of FFA1/GPR40 causes signal transduction through the PI3K =>PKB/Akt pathway, and the PLC =>PKC => p38/ERK–MAPK pathway, which causes phosphorylation and degradation of IκBα [181]. This leads to the NF-κB activation. As a consequence, the expression of IL-8/C–X–C motif chemokine ligand (CXCL)8 and cyclooxygenase-2 (COX-2) increases in neutrophils [181] and IL-6 increases in human cardiac microvascular endothelial cells [140].

## Consequences of the proinflammatory action of palmitic acid

### Palmitic acid increases proinflammatory cytokine production

PA can activate TLR4-mediated proinflammatory signaling pathways through the MyD88-dependent [92, 182] and MyD88-independent [88] activation of NF-κB. As a consequence, in macrophages and monocytes, there is an increased expression of cytokines such as IL-1β [85], TNF-α [86, 88], CCL2/monocyte chemoattractant protein-1 (MCP-1) [91, 130, 182], CCL4/macrophage inflammatory protein 1β (MIP-1β) [183] and increase in COX-2 [84, 136] and matrix metallopeptidase 9 (MMP-9) expression [85, 92]. PA also increases the LPS effect on the IL-1β [130], CXCL2 [131] and TNF-α production [130, 131]. However, PA can also induce TNF-α expression independently of TLR4 activation [54].

Nevertheless, in some cells, PA can increase the expression of proinflammatory cytokines. For example, CCL2/MCP-1 is secreted in adipocytes [86, 184]. In contrast, in TLR4-dependent manner, PA causes the increased expression of CCL2/MCP-1 and CXCL1 in pancreatic β-cells [185]. Also in the experiments on C2C12 myoblasts, PA causes TLR4-dependent increase in the IL-6 and TNF-α expression [102]. However, this effect is significant only after 6 h of incubation with PA. After 24 h, the importance of TLR4 is negligible.

In hepatocytes, PA causes IRE1α and JNK MAPK-dependent increase in the production of extracellular vesicles [62, 186]. These extracellular vesicles contain sphingosine-1-phosphate (S1P) [62, 187]. Also PA by the activation of the mixed lineage kinase 3 (MLK3) =>MAPK kinase (MKK)3/6 =>p38 MAPK =>signal transducer and activator of transcription 1 (STAT1) pathway increases the expression of CXCL10/IP-10 in extracellular vesicles [186, 188]. Activation of MLK3 may depend on DAG and PKC [189]. Other signaling molecule produced by hepatocytes under the influence of PA is tumor necrosis factor-related apoptosis-inducing ligand (TRAIL) [190]. It is a ligand for death receptor 5 (DR5)/TNFRSF10B that activates macrophages and NF-κB, which increases the production of IL-1β and IL-6 by these cells.

**Fig. 7:**
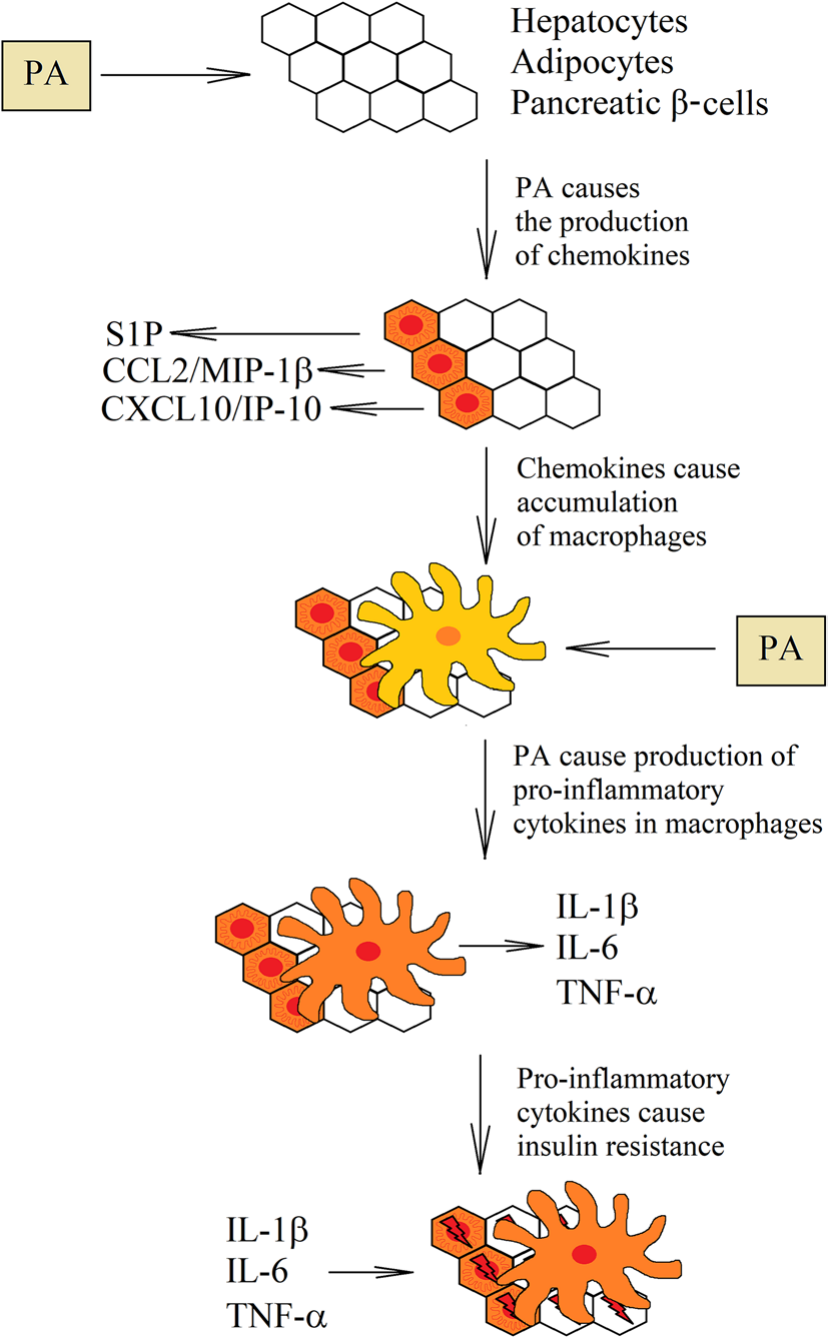
Role of macrophages in PA-induced insulin resistance. Healthy tissues contain a very small number of macrophages. However, under the influence of PA, hepatocytes, pancreatic β-cells and adipocytes begin to produce chamokines. This causes recruitment of macrophages to the liver, pancreas and adipose tissue. In turn, macrophages in these tissues begin to accumulate PA that causes inflammatory reactions. Increased production of proinflammatory cytokines results in insulin resistance in cells that are near the activated macrophages

The increased concentration of PA causes the production of chemokines by the cells. In particular, hepatocytes produce CXCL10/IP-10 [186, 188] and S1P [62, 187], adipocytes produce CCL2/MCP-1 [86, 184], and pancreatic β-cells the CCL2/MCP-1 [185]. All these chemokines cause recruitment of macrophages in the environment of these cells [64, 185, 191, 192]. Macrophages begin to form so-called crown-like structures in adipose tissue [64, 71, 73] and start to produce the proinflammatory cytokines involved in inflammatory responses and in insulin resistance [185, 191–193], such as TNF-α, IL-1β and IL-6 (Fig. 7).

### Consequences of high palmitic acid levels in the blood: insulin resistance

Under the influence of PA, macrophages accumulated in the tissues start to produce proinflammatory cytokines, which leads to insulin resistance (Tab. 1) [10]. The most important cytokines that cause insulin resistance are TNF-α [193, 194], IL-1β [195, 196] and IL-6 [190, 197].

**Table 1:** Mechanisms of PA-induced insulin resistance caused by the most important proinflammatory cytokines produced by macrophages

Cytokines	Cellular mechanisms for insulin resistance
IL-1β	Reduction of the IRS-1, IRS-2 and GLUT4 expression Apoptosis of pancreatic β-cells
IL-6	PKCδ-dependent phosphorylation of IRS-1 Increase of SOCS-3 expression
TNF-α	JNK–MAPK-dependent phosphorylation of IRS-1 Increase of PTP1B expression Increase of SOCS-3 expression IKK2-dependent increase in the S6K1 expression IKK-dependent phosphorylation of IRS-1

TNF-α causes insulin resistance in different ways. In hepatocytes, it causes the activation of JNK–MAPK signaling pathway, which phosphorylates insulin receptor substrate (IRS)-1 [198]. Also in myocytes [199] and adipocytes [200], there is a higher expression of protein tyrosine phosphatase 1B (PTP1B), which inhibits the effect of insulin receptor (IR). In adipocytes, the TNF-α leads to the increase of suppressor of cytokine signaling 3 (SOCS-3) expression [201]. This protein suppresses the action of cytokines, but also binds to the IR, disrupting the function of this receptor. SOCS-3 may also cause the proteolytic degradation of IRS-1 [202]. Furthermore, TNF-α causes an IKK2-dependent increase in the ribosomal protein S6 kinase (S6K1) expression in adipocytes and hepatocytes [203]. S6K1 phosphorylates IRS-1 causing disruption in IR signal transduction. However, IKK itself can also phosphorylate IRS-1, which causes insulin resistance in muscles [204].

Another important cytokine involved in PA-induced insulin resistance is IL-1β [195]. This cytokine reduces in adipocytes the expression of IRS-1, IRS-2 and glucose transporter 4 (GLUT4) [196, 205]. IL-1β is cytotoxic to pancreatic β-cells, causing apoptosis of these cells [195, 206]. However, it should not be forgotten that PA alone acts cytotoxically on pancreatic β-cells as well. It activates its FFA1/GPR40 receptor, which causes the release of Ca^2+^ ions from the ER [207]. This is the signal to start releasing insulin. However, the chronic activation of FFA1/GPR40 by PA causes the ER Ca^2+^ depletion, and consequently the ER stress and apoptosis of pancreatic β-cells [208].

In turn, IL-6 [77, 190, 197] causes the PKCδ-dependent phosphorylation of IRS-1 in muscles, which leads to insulin resistance [209]. Nevertheless, this effect is tissue specific. In hepatocytes, IL-6 works by inducing the expression of SOCS-3 [210].

It should be remembered that the production of proinflammatory cytokines by macrophages is only one of the possible mechanisms of PA-induced insulin resistance. Very important are also signaling pathways which are directly induced by PA. In particular, an increased amount of DAG causes phosphorylation of IRS-1 by PKCδ [211, 212], PKCε [213] and PKCθ [214, 215]. PKCθ and PKCε activate IKK, which also phosphorylates IRS-1 [213]. Another way leading to PA-induced insulin resistance is the increased amount of ceramides in the cell. This causes activation of PP2A and dephosphorylation of PKB/Akt [19, 216, 217]. Ceramides can also activate PKCζ, which binds and phosphorylates PKB/Akt [218, 219]. Beside its influence on PKC, PA leads to ER stress and activation of JNK–MAPK pathway, which phosphorylate IRS-1. This makes PA a very important factor contributing to insulin resistance [213, 220, 221].

## Conclusion

In obesity, a high concentration of PA causes insulin resistance which leads to diabetes. This state is called “diabesity”. There are many known PA-induced insulin resistance mechanisms. For instance, PA may induce signaling pathways that interfere with IR signal transduction. PA may also indirectly lead to insulin resistance by causing inflammatory reactions in macrophages. This results in increased proinflammatory cytokine production that causes insulin resistance in the cells closely located to activated macrophages. It seems that an important factor inducing the PA-mediated inflammatory response is the activation of TLR2- and TLR4-mediated signaling pathway.

However, in the cell, PA is metabolized to saturated DAG, ceramides and lysophosphatidylcholine. They can cause various effects, leading to inflammatory reactions. In particular, a high concentration of PA in macrophages leads to ER stress. Also, PKC activation by DAG and ceramides strengthens inflammatory reactions. Another way to induce inflammatory reactions by PA is to increase the ROS generation, which contributes to the disruption of the mitochondrial function.

Because PA can induce and enhance the inflammatory reactions through many mechanisms, it is very difficult to interfere with these reactions in people with obesity, mainly because of the fact that changes in one path are balanced by other proinflammatory pathways. Therefore, the best way to reduce the inflammatory response in obese patients is to reduce the free blood FA concentration. To do this, you should apply the appropriate diet or drugs that cause normalization of lipid metabolism.

## Abbreviations

ATF: Activating transcription factor
CHOP: C/EBP homologous protein
CCL: CC motif chemokine ligand
JNK: c-Jun NH_2_-terminal kinase
CD36: Cluster of differentiation 36
DAG: Diacylglycerol
DGAT: Diglyceride acyltransferase
ER: Endoplasmic reticulum
eIF2α: Eukaryotic initiation factor 2α
FABP4: Fatty acid-binding protein 4
GSK-3β: Glycogen synthase kinase-3β
IκB: Inhibitor of NF-kB
IKK: Inhibitor of NF-kB kinase
IRE1α: Inositol-requiring enzyme 1α
IRS: Insulin receptor substrate
IL: Interleukin
LPS: Lipopolysaccharide
MAPK: Mitogen-activated protein kinase
MUFA: Monounsaturated fatty acids
MyD88: Myeloid differentiation factor 88
NLRP3: NOD-like receptor pyrin domain containing 3
NF-κB: Nuclear factor κB
PPAR: Peroxisome proliferator-activated receptor
PI3K: Phosphatidylinositol 3-kinase
PUFA: Polyunsaturated fatty acids
PKB: Protein kinase B
PERK: Protein kinase RNA-like endoplasmic reticulum kinase
PKC: Protein kinases C
PP2A: Protein phosphatase 2A
ROS: Reactive oxygen species
RANTES: Regulated on activation, normal T cell expressed and secreted
SFA: Saturated fatty acid
SPT: Serine palmitoyltransferase
SIRT: Sirtuin
XBP-1s: Spliced X-box-binding protein-1
TXNIP: Thioredoxin-interacting protein
TRAF: TNF receptor-associated factor
TLR: Toll-like receptor
TAG: Triacylglycerol
TNF-α: Tumor necrosis factor-α
UCP2: Uncoupling protein 2
